# National Hospital for Paralysed and Epileptic

**Published:** 1886-12-25

**Authors:** B. Burford Rawlings

**Affiliations:** *Secretary and Director.* Queen Square, Bloomsbury


### THE EDITOR'S LETTER-BOX.

[Our Correspondents are reminded that prolixity is a great bar to publication, and that brevity of style and conciseness of statement greatly facilitate early publication.]

### NATIONAL HOSPITAL FOR THE PARALYSED AND EPILEPTIC.

My attention has been drawn to the statement in your "Diary," under the name of this Hospital, that "a portion of the beds are reserved free to the necessitous poor; the others are for paying patients."

Will you allow me to say that the free beds number nearly three-fourths of the whole (180), and that the others are for patients in straitened circumstances who contribute 21*s*. per week? Paying patients, in the ordinary acceptation of the term, we do not seek; our rules, strictly interpreted, exclude them. The only two remunerative patients the Hospital has had during the year were admitted under exceptional circumstances, at the request of, and out of courtesy to, members of the staff.

B. Burford Rawlings,
*Secretary and Director*.

Queen Square, Bloomsbury,
21st Dec. 1886.

